# Scirrhous Tumor of the Lip

**Published:** 1861-08

**Authors:** G. W. Powell

**Affiliations:** Childersburg, Alabama


### ARTICLE II.

*Scirrhous Tumor of the Lip.* By G. W. Powell, M. D., of Childersburg, Alabama.

In the outset of my practice, and among the first cases that I was called to treat, was that of a Scirrhous Tumor in the inferior or lower lip of a gentleman some fifty-two years of age. This gentleman, from early youth up to manhood, was troubled, during the winter seasons, with a fissure in the lip, which generally healed on the approach of the warm seasons. This phenomenon continued for a number of years, producing no pain or inconvenience, more than is usually experienced from like circumstances. But, in the course of time, a cartilaginous or horny-looking tumor began growing at this site. It scarcely ever reached a size larger than that of a grain of corn, before it would exfoliate or fall off; and then it would begin to grow again as it did at first, attaining a larger size at every exfoliation. The tumor thus continued to enlarge, until it reached a size inconvenient to the patient, when he began to pare it off with his knife, keeping it in due bounds, until it would again fall off. After every exfoliation, the lip would appear to be getting in a healthy condition, there being no unusual appearance, except some tenderness and redness. This tumor was painless, and gave no trouble, except its bulk, and some anxiety of mind in regard to its nature and final termination. The patient described this tumor as cutting like, and resembling horn or cartilage. Thus it would seem that there was a matrix (if I may be allowed the expression) embeded within the tissue of the lip, continually secreting and throwing out its matter, which, being exposed to external influences, became hard and cartilaginous. This phenomenon continued for some years, when, finally, this tumor exfoliated the last time, and the lip soon became ulcerated.

The character of the disease then became very much changed to the detriment and discomfort of the patient. While it had never been painful heretofore, he now suffered excruciating pain. At first the skin began to slough away, and finally the deeper tissue began to ulcerate, attended with lancinating pain and sleepless nights to the patient. As the disease progressed his general health became impaired, with almost a jaundiced appearance of the skin. The lip was eaten out into irregular cavities, filled with fetid and irritating matter, assuming the form of an open destructive cancer, and continuing its ravages until its removal by extirpation.

Sometime after, there appeared, just underneath the left eye, what is generally termed a cancer wart, but perhaps it was of the same nature and kind as that in the lip, except it was not very painful, and did not ulcerate. Occasionally crops of granulations would be thrown out, and there would be some improvement in the appearance of the ulcer, and in the feelings of the patient; but these granulations being too weak, or too contaminated to sustain themselves, were soon destroyed by the acrid secretions, and other causes common to their destruction.

Finally, all hopes of recovery, by resolution, being dispaired of, the patient made application to many physicians for treatment, in the course of a few years. Many physicians diagnosed the disease; some pronounced it a sloughing ulcer, others said it was a phagedenic ulcer, while another class affirmed that it was an eating cancer, etc. Many physicians, also, treated the case, but the treatment availed nothing in the end, notwithstanding it was as varied as was the physicians. One or two physicians, however, succeeded in healing the ulcer, with the exception of a small ulceration at the superior surface of the lip; but it would ulcerate again, and with greater rapidity than before, until it would reach its original stand; other treatment would produce an irritation and an aggravation to the disease, to the discomfort of the patient.

Such is the history of the case from its incipiency until February, 1858, when I first examined the case. At that time the patient was suffering excruciating pain, and passing sleepless days and nights without number, while the fetor from the lip was almost intolerable. The disease was spreading rapidly, and promised soon to be among the muscles and glands of the neck, a condition that would have been deplorable, and perhaps soon fatal to the patient. The induration already extended to the chin; the patient was pale and anæmic, and dispairing of all hopes of recovery. Being called upon to treat the case, I began the work; my object being, first, to elleviate pain, correct the fetor, and improve the general health; and, farther on, to cause the absorption or removal of the disease; and finally, to correct, as far as possible, the diathesis, and thereby prevent a return of the disease.

For the alleviation of pain, I used the ung. belladona or morphine, sprinkled upon the ulcer; Arg. Nit. in strong solution for a lotion twice a day, with chlorinated washes, creosote, etc., to correct the fetor. The constitutional remedies, consisting of mercurials, small portions combined with narcotics, and some of the preparations of iron, iodine, etc., as tonics and alteratives. Under this plan of treatment the pain was soon relieved, the general health began to improve, he passed the nights in sound sleep, and his appetite soon became craving. In a few weeks the patient was alarmed by a profuse hemorhage from the ulcer, produced from the use of the caustic, which, however, was soon arrested. In a few days after this, while having the ulcer dressed, it was discovered that some two or three small tumors lay upon the ulcer, nearly, or quite loose, and were removed. These tumors were described, by the patient and nurse, as being of a bluish color, and of a firm consistance. At that time there appeared to be but one tumor remaining, and near the angle of the mouth. This tumor was of larger dimensions than the others, and firmly adhered to the adjacent parts; its limits could not be determined with accuracy, as it gradually disappeared with the sound parts. The remaining portion of the ulcer began granulating, and healed rapidly until it was half healed, when of a sudden it was arrested, perhaps from the irritation or baneful influence of the remaining tumor. The lip became somewhat painful, and no appearance of healing. I then began the use of ung. iodine, Fowler's solution, creosote, etc., as lotions, and Donovan's solution as a constitutional remedy. Under this treatment the ulcer began to heal again, and did well for some time, when it was again arrested as before. I now dispaired of success with medicine, and determined (my patient being willing) to extirpate the entire mass, having carried him through a course of medicine of 8 months. During this time the general health was greatly benefited, the ulcer was free from pain, the induration extended very little below the ulceration, and I had great confidence in the success of an operation, notwithstanding I was opposed by the best of physicians.

On the 1st of October, 1858, the operation was performed by making a V incision, beginning near the angles of the mouth, cutting wide of the disease, until a point was reached entirely below the disease. Hemorhage was arrested by using one or two ligatures for the larger vessels, while the smaller ones were arrested by twisting. After hemorhage was arrested, the edges of the wound were brought together and secured by the quill suture. In a few days these sutures became loose, from sloughing and the force required to hold the edges together.

The wound was then again open, except a small portion at the vertex, that healed by the first intention. The chasm being so great it was impossible to reunite the edges by adhesive plaster, and to use sutures again was impracticable, I

therefore made incisions in the cheeks, in order that the edges might approach more easily. The edges of the wound were then brought together and secured by adhesive plaster, leaving the incisions in the cheeks to heal and fill up by granulation. The cold water dressing was used for some time, and lastly, the simple cerate, until entirely healed. He now has a very respectable-looking mouth, free from pain, filth and fetor, comfortable nights, etc. After the operation, I again put him upon the use of Donovan's solution, to farther correct the constitutional tendency, and thereby prevent a return of the disease. Under a coutinuation of this treatment, his health improved very rapidly, the tumor under the eye nearly disappeared, and he considered himself a sound man. He was able to attend to his business, and said he felt younger and stouter than he had for years.

His good health continued until July, 1860—being nearly two years—at which time he was seized with symptoms of cholera morbus, and after vomiting several times he became insensible, the bowels ceased to act, but the nausea and vomiting contiuued. He seldom answered any questions, and when he attempted it, they were so incorrect as to prove that there was great mental derangement. He never refused to take anything that was offered to him as medicine or nourishment. In a few hours after the attack I was sent for, and found him in the condition above stated. I learned the manner of the attack, and thought it was cholera morbus, and thought that it was in consequence of the nausea that he refused to speak. The pulse beat 90 per minute; not full and strong, but rather weak. I was not satisfied, however, in regard to the diagnosis; the inability to speak gave me some uneasiness of mind. I thought, however, that his case clearly indicated the use of mercurials, and therefore began the use of calomel and opium, in small doses; but his stomach rejected everything that was given him during the night. I then applied sinapisms to the epigastrium, and continued the calomel. The vomiting continued 36 hours, the pulse remaining the same. I continued to give him calomel, and at the same time gave injections until the bowels acted. He still refused to speak, except occasionally he would murmur that he was sick all over, and nothing more could be gotten from him for the next twenty days. The pulse became slower and fuller, with such a volume as I had never felt before. I was fearful that he was appoplectic, and sent for a consulting physician; he strengthened me in my views. But the cause was not of easy diagnosis; perhaps he was predisposed and the vomiting acted as the exciting cause. The pulse now 80 per minute, very full and strong, more so than any I had ever witnessed. I gave him a full bleeding from the arm, and for a short time the pulse was nearly natural, but in a few hours was nearly or quite as full as before. I had some fears in bleeding him again from the arm, and, therefore, applied cups to the nape of the neck, elevated the head and shoulders and applied cold water to the head, and continued the calomel in broken doses. On the fifth day of the disease I noticed symptoms of hemiplegia in the right side, which continued until it became of the severest form. I shaved the head and blistered the entire scalp, and continued the mercurial, as there was still no symptoms of ptyalism. In the course of another day or two there was restlessness and jactation, the temperature of the paralyzed limbs were greatly reduced and wasting, I therefore had them well sponged with stimulating liniments.

By the eighth day the pulse beat 70 per minute, and very weak, skin pale and rather cool, perspiration clammy, paralytic limbs wasting, the muscles flaccid, and greatly reduced in temperature, and the breathing twelve per minute. Every symptom appeared to me to indicate a speedy dissolution, unless promptly relieved. I applied mustard to the wrists and ankles, and had the extremities well sponged with ammoniated liniments, at the same time administering quinine and brandy in small portions. In a few hours the breathing became faster, and the pulse fuller and stronger, no other improvement being discoverable. The above treatment was continued till the 12th day of the disease, when ptyalism was established. The mercurial was then reduced in quantity and frequency, the tonics and stimulants being continued, and the blister kept discharging by the use of ung. antim.

There was very little improvement until the 18th, when the symptoms appeared more favorable, and in two or three days more he became conscious of all that was going on around him, but could not recollect anything more than a few minutes, and was greatly surprised at the paralysis, not knowing that he had been ill.

Convalescence was very rapid, and in ten days more he was able to walk with the assistance of his cane. In the meantime the blister was kept discharging, and mercurials, in doses large enough to keep up the salivation, and sponging the extremities with stimulating liniments. Headache was incessant for some 15 days longer, but gradually disappeared.

Again, on the 25th of October, he was seized with appoplexy; in a few minutes after the attack, I found him in a state of insensibility, pulse slow, but full and strong, and the breathing slow and stertorous. I gave him a full bleeding, elevated the head and shoulders and applied cold water to the head, and administered a dose of calomel. In a few hours he became conscious, complained of headache and an increase of paralysis.

Again, on the 2nd of December, he had another attack; but it was much lighter than the preceding one. I administered the same treatment as before, and again he was very soon canvalescing. Since that time he has enjoyed excellent health, except the paralysis and dimness of vision which has attended him since the first attack.

For two months past I have him on the use of strychnia, which seems to be relieving the paralysis and strengthening the vision. Some physicians have condemned my course in extirpating the disease, but I feel confident that I pursued the right course, and should another case present itself to me, I would pursue the same course.

If it was the suppression of a constitutional drain that caused the appoplexy, I would propose to establish another by the seton, issue-pea, or any other means that would establish one. Such a drain would be more agreeable to the patient, more cleanly, and less injurious to the system, than a foul, fetid and destructive cancer, let it be situated where it might, especially if the cancer was situated in the mouth, as it was in this case.

